# Preclinical Evidence of *Curcuma longa* and Its Noncurcuminoid Constituents against Hepatobiliary Diseases: A Review

**DOI:** 10.1155/2020/8761435

**Published:** 2020-07-28

**Authors:** Soyeon An, Eungyeong Jang, Jang-Hoon Lee

**Affiliations:** ^1^Department of Clinical Korean Medicine Graduate School, Kyung Hee University, 26 Kyungheedae-ro, Dongdaemun-gu, Seoul 02447, Republic of Korea; ^2^Department of Internal Medicine, College of Korean Medicine, Kyung Hee University, 26 Kyungheedae-ro, Dongdaemun-gu, Seoul 02447, Republic of Korea; ^3^Department of Internal Medicine, Kyung Hee University Korean Medicine Hospital, 23 Kyungheedae-ro, Dongdaemun-gu, Seoul 02447, Republic of Korea

## Abstract

Hepatobiliary disease currently serves as an important public health issue due to the fact that it is one of the major causes of death among economically active individuals and can easily progress to chronic diseases. Despite the development of vaccines and numerous drugs, a definite treatment remains lacking owing to different stages of the disease itself, its intricate pathogenesis, an effect uncertainty for long-term use, resistance, and side effects. *Curcuma longa* (*C. longa*), which belongs to the family Zingiberaceae and the genus *Curcuma*, has long been used not only as spice for curry or dye but also as a constituent of herbal formula for the treatment of different diseases due to its bioactive activities. Recently, many studies on the experimental results of *C. longa* have been published relative to hepatobiliary diseases such as fatty liver, hepatitis, cirrhosis, and tumors. Therefore, in this review, we aimed to summarize the pharmacological effects and underlying molecular mechanisms of *C. longa* and its four compounds, *β*-elemene, germacrone, ar-turmerone, and bisacurone, against hepatobiliary diseases. *C. longa* exhibited antioxidant, hepatoprotective, antisteatotic, anti-inflammatory, antifibrotic, antitumor, and cholagogic effects by regulating apoptosis, CYP2E1, Nrf, lipid metabolism-related factors, TGF-*β*, NF-*κ*B, CYP7A1, and so on. In particular, *β*-elemene could be an attractive compound owing to its remarkable hepatoprotective, anti-inflammatory, antifibrotic, and antitumor activities. Altogether, the present review provides a preclinical basis for the efficacy of *C. longa* as an effective therapeutic agent for the prevention and treatment of hepatobiliary diseases, despite the need for further studies to establish the extraction conditions and separation of active constituents with high bioavailability, and warrants further evaluation in clinical trials.

## 1. Introduction

Hepatobiliary disease includes a wide spectrum of hepatic and biliary disorders which are caused by damages induced by toxic chemicals, drugs, alcohol consumption, viral infection, carcinogen, and obesity. Recently, acute and chronic diseases of the liver and biliary system have become a major public issue as current hepatobiliary diseases such as nonalcoholic fatty liver disease, drug-induced liver injury, hepatocellular carcinoma, and gallbladder, and biliary tract cancers are closely related to recent lifestyle changes [1]. Both males and females are vulnerable to different hepatobiliary diseases despite the sex disparity that exists in the prevalence of autoimmune diseases, gallstones, and hepatocarcinoma (HCC) and alcohol metabolism [2, 3]. Unfortunately, most hepatobiliary diseases are diagnosed at the advanced stage where they have already progressed as most patients experience few signs or symptoms. Hence, the prevalence, mortality, and the health care burden of hepatobiliary diseases are steadily increasing. Approximately 2 million liver-related deaths occur every year worldwide [4]. However, the true global burden could be higher than expected. Although there has been evident breakthrough with curative agents over the last decades to manage hepatobiliary diseases, an optimal preventive and therapeutic strategy has yet to be established because of the several stages of the disease itself, complicated pathogenesis, low efficacy at long-term use, drug resistance, and undesirable side effects.

Preventing and treating hepatobiliary diseases with medicinal plants has had a long history. To date, many herbal medicines including *Silybum marianum* (milk thistle), *Glycyrrhiza glabra*, *Phyllanthus amarus*, *Artemisia capillaris, Lysimachia christinae,* and *Lonicera japonica* have been demonstrated to exhibit beneficial effects in managing different indications related to liver and biliary dysfunctions without harmful side effects [5, 6]. From the hepatoprotective and therapeutic viewpoints, *Curcuma longa* (*C. longa*) deserves clinical attention. According to the TCM theory, the radix (Yujin in Chinese) and rhizoma (Jiang Huang in Chinese) of *C. longa* are commonly attributed to the liver meridian [7]. Accumulating data have emphasized the pharmacological effects of *C. longa* for resolving pathological disorders in the hepatic, gallbladder, and biliary system. In addition, curcumin, a representative compound from *C. longa*, was introduced as a promising candidate that exhibits anti-inflammatory, antifibrotic, and antitumor activities which contribute to the treatment of viral hepatitis, stone, fibrosis, nonalcoholic steatohepatitis (NASH), primary sclerosing cholangitis (PSC), HCC, cholangiocarcinoma, etc. [8]. Due to the low absorption and bioavailability of curcumin, assuming that the effects of *C. longa* are largely attributed to the efficacy of curcumin might be misleading.

To date, a review article summarizing the preclinical evidence of *C. longa* or its active constituents, besides curcumin, against hepatobiliary diseases has not been published. Herein, we aimed to demonstrate available experimental results and the molecular mechanism of *C. longa* and its four constituents that are related to the prevention and treatment of hepatobiliary diseases.

## 2. Pharmacological Activities of *C. longa*

Liver disease represents a group of disorders characterized by stages of progression from steatosis and hepatitis to cirrhosis and cancer. This diverse spectrum of hepatic diseases is markedly associated with the pathophysiology of the biliary system [9]. According to the pathological stages of hepatobiliary disease, the pharmacological activities of *C. longa* can be generally classified into hepatoprotective, antioxidant, antisteatotic and antilipidemic, anti-inflammatory, antifibrotic, antitumor, and cholagogic effects.

### 2.1. Hepatoprotective Effect

The liver is a major organ in the management of metabolism, detoxification, and immune function. Moreover, it has a remarkable role of regenerating injured hepatocytes and restoring these cells to their original state. However, long-term exposure to different factors, such as virus, drugs, alcohol, and obesity, causes the liver to lose its restoration ability, eventually resulting in hepatitis, liver cirrhosis, and cancer. Although strong radical scavengers are widely applied to treat different liver diseases, the clinical efficacy of antioxidants is yet to reach a consensus [10]. Thus, the therapeutic agents that manage a variety of hepatic diseases need to exhibit hepatoprotective and antioxidant effects [11].

Carbon tetrachloride (CCl_4_) is a representative toxic material that induces severe liver injury, which may result in hepatic cirrhotic changes. In rodent models, oral administration and intraperitoneal injection of CCl_4_ increased the levels of the biochemical indicators (alanine aminotransferase (ALT), aspartate aminotransferase (AST), lactate dehydrogenase (LDH), alkaline phosphatase (ALP), and total bilirubin (TB)) and the hepatosomatic index with hepatic lobular disorganization. *C. longa* reversed these abnormal measures and improved the histological changes [12–24]. The rhizomes of *C. longa* lowered the levels of serum AST, ALT, and ALP, hepatosomatic index, and mortality in rats with diethylnitrosamine- (DEN-) induced carcinogenic liver injury [25–27]. Furthermore, in the presence of thioacetamide (TAA) and aflatoxin, which are toxic carcinogens, *C. longa* recovered serum AST, ALT, protein, and albumin levels and decreased the hepatosomatic index in Sprague Dawley (SD) rats and broiler chickens, respectively [28, 29].

In addition to the above toxic substances, *C. longa* exerted hepatoprotective effects against alcohol [30–34], drugs [35–38], and NASH-induced methionine-choline deficient (MCD) diet [39]. The methanol extract of the rhizomes of *C. longa* exhibited a stronger cell viability in normal primary hepatocytes than curcuminoids [30], which suggests the potential of the noncurcuminoid ingredients to relieve alcohol toxicity. In drug-induced liver injury (DILI) caused by anti-inflammatory analgesic [35], antituberculosis [36], anticancer [37], and immunosuppressant [38] drugs, *C. longa* normalized the levels of AST, ALT, ALP, and TB, which are key indicators of the DILI assessment tool of the Council for International Organizations of Medical Sciences (CIOMS). *C. longa* improved histological liver injury and the serological measures against acetaminophen, a well-known DILI-inducing drug, in male SD rats [35].

*C. longa* exerted hepatoprotective properties against liver injury induced by heavy metals, such as lead [40] and mercury [41], and toxic pesticides, such as carbofuran [42] and endosulfan [43], by lowering the serum levels of AST, ALT, ALP, gamma-glutamyl transpeptidase (GGT), and TB, improving hepatic protein synthesis, and preventing toxicity-induced weight loss in rats and chickens. Specifically, oral supplementation of *C. longa* (500 mg/kg daily for 28 days) led to a significant reduction of the elevated AST, ALT, and ALP in Wistar albino rats, which are abnormal markers associated with the hepatocellular damage. Treatment with *C. longa* attenuated a significant increase of lipid peroxidation (LPO) and elevated the level of glutathione (GSH), which suggest that the possible molecular mechanism of pharmacological effects of *C. longa* against lead-induced hepatotoxicity might be involved in reducing oxidative stress [40]. In addition, the ethanol extract of the rhizomes of *C. longa* upregulated the expression of hepatic microsomal proteins that play a critical role in detoxification, which may contribute to its beneficial activity against HgCl_2_-induced hepatotoxicity in SD rats [41].

Therefore, *C. longa* protected the liver from different factors, such as chemicals, drugs, alcohol, heavy metals, and pesticides, which may increase the risk of liver injury, by inhibiting apoptosis and the normalization of serological and histological changes. Further studies focusing on the medicinal parts, extraction, and the chemical constituents of *C. longa* related to its hepatoprotective activities are required to strengthen its uses in the clinical settings.

### 2.2. Antioxidant Effect

Although blood tests and image inspections of patients with liver diseases reveal normal ranges, oxidative damage is frequently observed in the liver. For example, serum AST or ALT levels were normal or slightly elevated in obese fatty liver patients. However, there was a definite change in the oxidative stress markers of hepatic tissue [44]. Hence, the antioxidant effects of *C. longa* can play a crucial role in the management of hepatobiliary diseases because oxidative stress is closely associated with hepatic steatosis, inflammation process, cirrhosis, and tumorigenesis.

CCl_4_ [12–14, 16–19, 23], DEN [25], TAA [28], p-dimethylaminobenzaldehyde (p-DAB) [45], and benzopyrene [46] are the main reagents used to induce intrahepatic oxidative stress in most experiments performed to investigate the antioxidant effects of *C. longa*. Most of these chemicals are metabolized in the hepatic endoplasmic reticulum, and their metabolites lead to protein and lipid peroxidation, depletion of antioxidant enzymes, and triggering of hepatic necrosis by covalently binding with nuclear DNA [47]. First, *C. longa* increased the production of the antioxidant factors, such as superoxide dismutase (SOD), catalase (CAT), glutathione peroxidase (GSH-Px), and glutathione reductase (GR) in the liver of rodents, which were depleted by the above chemicals. In addition, *C. longa* regulated the intrahepatic malondialdehyde (MDA) levels, one of the most popular biomarkers of oxidative stress in rats administered TAA. Furthermore, the 95% ethanol extract of the rhizomes of *C. longa* reversed the levels of hepatic nitrotyrosine (a biological marker for protein oxidation) and urinary 8-hydroxy-2-deoxyguanosine (8-OH-dG), indicating DNA oxidative damage, as effective as silymarin in SD rats [28].

*C. longa* optimized the level of intrahepatic antioxidant molecules and reduced the production of lipid peroxidation against oxidative damage caused by alcohol [19, 33, 34, 39], drugs [17, 35, 37, 38, 48], MCD diet [39], pesticides [42, 43], heavy metals [40, 41], and iron [49]. The inhibition of intrahepatic cytochrome P450 2E1 (CYP2E1) by *C. longa* contributed to its antioxidant effects against alcohol-induced oxidative stress in C57BL/6 mice [33]. *C. longa* facilitated the reduction of Fe^3+^ ion in male New Zealand rabbits [49], which could contribute to the protection of liver tissue from oxidative damage caused by excess iron, during iron overload-induced liver injury.

Altogether, *C. longa* might exhibit strong antioxidant activities against the precursors, causing oxidative stress in the liver, such as chemicals, carcinogens, alcohol, drugs, pesticides, heavy metals, and iron, and two molecular mechanisms can be involved in its action. First, *C. longa* could markedly prevent and inhibit the overproduction of free radicals and lipid peroxides in the hepatic and gall bladder tissue by mediating a significant amount of CYP2E1 expression. CYP2E1 often generates reactive oxygen species, such as the superoxide anion radical and hydrogen peroxide, and it is frequently activated in chronic liver diseases [50]. Hence, further studies focusing on the antioxidant activities of *C. longa* via the regulation of intrahepatic CYP2E1 must be implemented to enable the use of this herb to treat hepatobiliary diseases. Second, *C. longa* exhibited some strengths in increasing the amount of antioxidant materials, and one of the underlying mechanisms might be the transcriptional regulation of nuclear factor erythroid 2-related factor 2 (Nrf2) targets. The rhizomes of *C. longa* increased intrahepatic Nrf2 levels in a dose-dependent manner against CCl_4_-induced injury. Additionally, antioxidant enzymes, such as SOD and CAT, were upregulated. Its efficacy to remove free radicals and lower lipid peroxidation is comparable to that of butylated hydroxytoluene (BHT) [12] and vitamin C [48], respectively. Moreover, *C. longa* lowered the intrahepatic accumulation of lipid peroxides and activated the antioxidant defense system without causing toxicity in normal Wistar rats [51]. Hence, *C. longa* might be employed to manage different hepatobiliary diseases owing to its antioxidant activities.

### 2.3. Antisteatotic and Antilipidemic Effects

The liver is in charge of lipid homeostasis by controlling the uptake and breakdown of dietary fatty acids for energy production, synthesizing de novo lipogenesis, or excretion from the liver [52]. Simple hepatic steatosis is defined as the intrahepatic accumulation of at least 5% of triglyceride (TG) of liver weight due to fatty acid surplus. Intrahepatic fat infiltration is itself the cause of NASH and accelerates the oxidative damage of hepatocytes, which makes the liver vulnerable to inflammation or fibrosis progression. In addition, hepatic steatosis is regarded as an independent risk factor of metabolic syndromes, such as insulin resistance, dyslipidemia, and cardiovascular diseases, and it has been reported to be more closely associated with metabolic syndromes than obesity [53].

The antisteatotic effects of *C. longa* were investigated in rodent models induced by CCl_4_ [13, 14, 21, 23], high-fructose diet [54, 55], and high-fat diet [54, 56, 57]. *C. longa* decreased the levels of TG contents, total cholesterol, and low-density lipoprotein (LDL) in the liver tissue, and hepatic histological findings by Oil Red O staining were consistently improved. Regarding the antisteatotic effects of *C. longa*, its water extract at 250°C impeded the uptake of fatty acids into the liver by suppressing the mRNA expression of CD36 and fatty acid transport protein (FATP) in C57BL/6 mice. In addition, it inhibited intrahepatic lipid synthesis via the regulation of sterol regulatory element-binding protein-1c (SREBP-1c), fatty acid synthase (FAS), and acetyl CoA carboxylase-1 (ACC) mRNA levels. Furthermore, it promoted lipolysis partially through the regulation of AMP-activated protein kinase (AMPK), peroxisome proliferator-activated receptor-*α* (PPAR-*α*), and carnitine palmitoyltransferase-1 (CPT-1) mRNA levels [56]. Particularly, the rhizomes of *C. longa* facilitated the secretion of lipids from the liver into blood via the increase in hepatic PDI [13] and betaine [54] expression in male SD rats.

It is important to modulate the lipid contents that circulate in the blood and the inhibition of intrahepatic lipid accumulation for the treatment of hepatic steatosis. *C. longa* increased the level of serum high-density lipoprotein (HDL) and lowered the level of serum LDL, TG, and total cholesterol against high-fat diet [21, 57–59], carcinogen [26, 29, 41, 45, 46], ethanol [34], and pesticides [42, 43]. In addition, *C. longa* decreased the regions of aortic fatty streak in rabbits with atherosclerosis [58]. The modulatory effects of *C. longa* against serum lipids might inversely aid in the reduction of lipid inflow into the liver tissue.

In conclusion, *C. longa* can be developed as an important agent to treat fatty liver diseases, which account for a large portion of hepatobiliary diseases. In particular, the water extract of *C. longa* was found to exhibit strong antisteatotic and hypolipidemic effects, which were involved in the pharmacological mechanisms related to lipid metabolism.

### 2.4. Anti-Inflammatory Effect

Inflammation has a close interrelationship with oxidative stress and steatosis in the pathogenesis of hepatobiliary diseases. The management of inflammation is crucial in the treatment of hepatobiliary diseases because hepatitis can be regarded as a stage prior to the development of cirrhosis or cancer [60, 61].

The anti-inflammatory effects of *C. longa* were mainly elucidated by a significant reduction in hepatic and serum levels of tumor necrosis factor-*α* (TNF-*α*), which was elevated by drugs [35], ethanol [32], MCD diet [39], and TAA [28]. *C. longa* markedly decreased the hepatic interleukin-6 (IL-6) value in C57BL/6 mice administered alcohol [32] and an MCD diet [39], which are models that demonstrate the important role of IL-6 in the development of alcoholic or nonalcoholic fatty liver into cirrhosis or cancer [62]. *C. longa* improved the major hallmark of the inflammation-associated histological findings, such as periportal inflammatory cell infiltration [19, 36, 42], hepatic vascular congestion [34, 41], F4/80-positive macrophages [39], and mononuclear cellular infiltration [34].

These pharmacological activities of *C. longa* were mainly based on the decrease in inflammatory cytokine production by the inhibition of lipid peroxidation through its antioxidant actions. In a liver injury model induced by valproic acid and paracetamol, the water extract of *C. longa* lowered the intrahepatic MDA levels by increasing antioxidant enzymes, resulting in the suppression of TNF-*α* activity in the liver of male SD rats [35]. Similarly, *C. longa* augmented SOD and CAT levels and reduced the levels of intrahepatic MDA and serum TNF-*α* in a TAA-induced liver cirrhosis model [28].

Therefore, *C. longa* might exhibit strong anti-inflammatory effects against inflammatory reactions in the liver. However, its efficacy should be investigated in experimental models that mimic cholangitis or cholecystitis because curcumin alleviated sclerosing cholangitis in mice [63].

### 2.5. Antifibrotic Effect

Fibrosis involves excess extracellular matrix (ECM), such as collagen and fibronectin, and this production is initiated by the activation of hepatocellular stellate cells into myofibroblasts in the process of repairing damaged hepatocytes. Fibrosis can result in reduced liver function, hepatocyte necrosis, decreased intrahepatic microcirculation, and even cirrhosis or cancer [64]. However, because fibrosis can be reversed unlike liver cirrhosis, active management of liver fibrosis is required.

First, *C. longa* was found to reduce the amount of collagen, which was accumulated in the liver tissue. The hot water extract of the rhizomes of *C. longa* inhibited the excessive accumulation of alpha-1 type I collagen and the sirus-red positive area in the liver of mice fed an MCD diet [39]. Second, *C. longa* enhanced the expression of enzymes promoting collagen degradation in the liver tissue by inhibiting tissue inhibitor of metalloprotease (TIMP)-1, which blocks the activity of matrix metalloprotease-2 (MMP-2) [39]. Third, *C. longa* could markedly suppress hepatic alpha-smooth muscle actin (*α*-SMA), a specific protein marker of hepatic stellate cells (HSCs) contributing to the progression of liver cirrhosis [39]. The antifibrotic activities of *C. longa* may be exhibited via two molecular mechanisms—the blockade of transforming growth factor-*β* (TGF-*β*) signaling pathway [28, 39] and the induction of apoptosis of HSCs [65].

Hence, *C. longa* reduced collagen accumulation, accelerated the decomposition of the ECM, and inhibited the activity of proteins involved in the progression of liver cirrhosis. In addition, the rhizomes of *C. longa* shortened the prolonged prothrombin time in TAA-induced SD rats [28]. The effects of *C. longa* against liver cirrhosis and its complications, such as delayed coagulation, should be investigated in further research. Regarding TGF-*β* signaling suppression and proapoptosis of *C. longa* against hepatic fibrogenesis, PPAR-*γ* activation can hinder binding to TGF-*β* and induce apoptosis of HSCs. Such findings suggest that *C. longa* could act as a PPAR-*γ* agonist in the treatment of hepatobiliary diseases.

### 2.6. Antitumor Effect

Most experimental studies demonstrating the antitumor effects of *C. longa* against hepatobiliary system were based on HCC cell models or animal models induced by carcinogens of HCC. *C. longa* inhibited the proliferation of HepG2 cells by inducing apoptotic changes [66]. In addition, the rhizomes of *C. longa* lowered the HCC incidence rate [27] and the level of the serum tumor marker, *α*-fetoprotein [26], in DEN-stimulated SD rats. In parallel, histological findings were characterized by the reduction in the number and size of GGT-positive hepatocytes, which are important in tumorigenesis in SD rats induced by DEN [26, 27]. Furthermore, *C. longa* impeded tumor angiogenesis by decreasing the level of serum vascular endothelial growth factor (VEGF) in DEN-induced Wistar rats [25]. In p-DAN and phenobarbital-induced rat models, the ethanol extract of the rhizomes of *C. longa* inhibited liver carcinogenesis by suppressing the hepatic expression of p53 and Bcl-2 and prevented cancer metastasis by disturbing hepatic MMP activity [45].

With regard to cholangiocarcinoma, *C. longa* displayed antitumor effects by upregulating the apoptosis of RMCCA1 cells via the activation of the mitogen-activated protein kinase (MAPK) signaling pathway [67].

In summary, *C. longa* might exert its antitumor effects on tumor markers, angiogenesis, and tumorigenesis through cell cycle arrest, Bcl-2 suppression, and MAPK signaling activation. Its anticancer activities can be applied to treat both HCC and bile duct cancer. Additionally, unlike conventional anticancer drugs, *C. longa* may have fewer side effects, such as weight loss and anorexia. Through further studies, *C. longa* might be demonstrated as a potent anticancer herb for the treatment of hepatobiliary cancer.

### 2.7. Cholagogic Effect

Cholestasis, a condition involving a decrease in bile flow, is caused by multiple factors, including infection, alcohol, drugs, tumor, and autoimmunity. Cholestasis can cause damage to organelles and cell membranes in hepatocytes and dysfunction in the hepatobiliary system, eventually resulting in symptom manifestation, such as jaundice, xanthoma, and itch [68]. Although antibiotics/antivirals and immunosuppressants are used to treat infectious cholestasis and autoimmune cholestasis, respectively, ursodeoxycholic acid (UDCA) is currently recommended as the primary therapeutic drug to improve clinical symptoms and bile flow [69]. However, the development of drugs to treat cholestasis and prevent its complications is urgently required as the effects of UDCA remain limited.

*C. longa* elevated the total amounts of bile acids and bile secretion by activating the bile excretion pump in a concentration-dependent manner [41, 70, 71]. In particular, the water extract of the radix of *C. longa* increased the level of serum total bile acids by UDP-glucuronyl transferase activity for cholesterol excretion from the liver in high-fat diet rat models. This result is accompanied by the increased TG discharge to stool and decreased serum lipid contents [72]. Similarly, the water extract of the rhizomes of *C. longa* activated the cholesterol 7*α*-hydroxylase (CYP7A1) enzyme converting cholesterol into bile acids in SD rats fed a high-fat diet [57].

In summary, *C. longa* exhibited cholagogic effects by increasing the production and secretion of total bile acids in high-fat diet-induced rats. However, there are no animal models induced by bile duct ligation or related to liver cirrhosis for evaluating the efficacy of *C. longa*. Therefore, different models stimulated by drugs, alcohol, autoimmune inflammation, viral infections, etc., are required to assess the pharmacological effects of *C. longa* in the treatment of cholestasis-induced dysfunctions.

## 3. Pharmacological Activities of Active Compounds Isolated from *C. longa*

**Figure 1:**
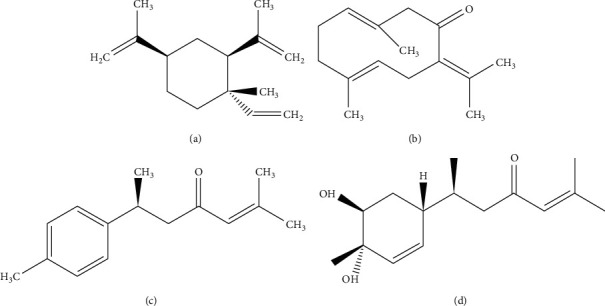
Four noncurcuminoid compounds obtained from *C. longa*. (a) *β*-Elemene (C_15_H_24_, molecular weight (MW) of 204.34 g/mol). (b) Germacrone (C_15_H_22_O, MW of 218.34 g/mol). (c) Aromatic turmerone (ar-turmerone, C_15_H_20_O, MW of 216.32 g/mol). (d) Bisacurone (C_15_H_24_O_3_, MW of 252.354 g/mol).

The main constituents that contribute to the bioactive effects of *C. longa* on hepatobiliary disease are largely grouped as curcuminoids and noncurcuminoids [73]. Curcuminoids, which are lipophilic polyphenols, account for approximately 5% of *C. longa*. However, their efficacy has been constantly reported. In particular, curcumin, the principal curcuminoid, has demonstrated antioxidant, anti-inflammatory, antiviral, antifibrotic, and anticancer effects against hepatobiliary diseases [8]. However, extensive studies focusing on noncurcuminoids, such as elemene, germacrone, turmerone, and bisacurone, have recently accumulated [74]. Therefore, this review presents the pharmacological actions of the 4 noncurcuminoid constituents from *C. longa* which might have therapeutic effects against hepatobiliary diseases (Figure 1).

### 3.1. *β*-Elemene

*β*-Elemene (Figure 1(a), C_15_H_24_, 204.34 g/mol) is a member of the elemene sesquiterpenoids derived from the essential oil of *C. longa* [73]. Its compound exerted beneficial activities against various types of tumor, such as lung cancer, breast cancer, prostate cancer, cervical cancer, gastric cancer, and sarcoma cancer [75]. Similarly, most experimental studies of *β*-elemene targeting hepatobiliary diseases have been related to cancer. In mice models transplanted with human HCC cell lines (H22 [76] and MHCC97H cells [77]), *β*-elemene reduced the weight and volume of the tumor tissue. In particular, *β*-elemene enhanced the sensitivity of the anticancer drugs by increasing the level of copper transporter 1, aiding in the uptake of oxaliplatin into liver cells [77]. Based on the underlying mechanisms of its anticancer effects, *β*-elemene inhibited the proliferation of cancer cells by increasing histone H1 protein in H22 cells [76], and its treatment induced G2/M arrest and apoptosis of HepG2 cells by augmenting Fas and FasL mRNA and proteins on the cellular surface [75].

In addition to anticancer effects, *β*-elemene exerted anti-inflammatory effects by downregulating hepatic CD14 expression and suppressing serum TNF-*α* and endotoxin in CCl4-induced rats [78]. *β*-Elemene lowered the serum AST and ALT levels in rats with CCl4-induced liver damage [79]. In addition, *β*-elemene decreased dendrites and increased the vesicular structure of LX-2 cells, thereby reducing liver fibrosis [80]. Similarly, *β*-elemene injection was found to lower hepatic collagen deposition in CCl_4_-induced rats, which might be caused by the reduction in serum angiotensin-II level and hepatic angiotensin-II type 1 receptor mRNA level [79].

In summary, *β*-elemene might be one of the key active constituents of *C. longa* for the treatment of hepatobiliary diseases because it exerted inhibitory effects against liver injury, inflammation, and fibrosis in different experimental models.

### 3.2. Germacrone

Germacrone (Figure 1(b), C_15_H_22_O, 218.34 g/mol) belongs to the volatile sesquiterpene family from *C. longa*. The pharmacological activities of germacrone in hepatobiliary diseases can be classified into two categories, namely, hepatoprotective and anticancer effects. Two *in vivo* experiments in Japan demonstrated that the intake of germacrone reversed the increase in the serum level of AST and ALT induced by D-galactosamine (D-GalN) in mice [81, 82]. In human hepatoma cell lines, such as HepG2 and Bel7402, germacrone inhibited cellular proliferation by upregulating the apoptosis index. In particular, germacrone exhibited the induction of apoptosis similar to the control group in the normal liver cell line, LO2 cells, and significantly induced the apoptosis of HepG2 cells. These findings suggest that germacrone displays less toxic hepatoprotective effects and has strengths as it is only toxic to tumor cells [83].

Therefore, similar to *β*-elemene, germacrone is expected to not only possess hepatoprotective effects but also anticancer effects. Future research on biliary tract cancer and liver cancer must be conducted to identify its high potency against cancer with minor impacts on normal cells.

### 3.3. Ar-Turmerone

Aromatic turmerone (ar-turmerone, Figure 1(c), C_15_H_20_O, 216.32 g/mol) was introduced as a representative active compound of *C. longa* with curcumin [84]. A study conducted in Egypt reported that *C. longa* comprised more ar-turmerone than curcumin [66].

Ar-turmerone exhibited hepatoprotective, anticancer, and cholagogic effects against hepatobiliary diseases. Ar-turmerone improved the ethanol-induced reduction in the cell viability of hepatocytes isolated from SD male rats [30]. In normal Wistar rats, intraduodenal injection of ar-turmerone increased the volume of total bile acids and bile secretion [71]. In addition, ar-turmerone caused significant inhibitory effects on the cell proliferation of three HCC cells, namely, HepG2, Huh-7, and Hep3B. In particular, ar-turmerone induced both regulation of Bax and p53 upregulated modulator of apoptosis (PUMA) of the intrinsic apoptotic pathway and caspase activation of the extrinsic apoptotic pathway [85].

In recent studies, ar-turmerone enhanced the immune system by elevating the number of monocytes in peripheral blood [86] and exhibited antiangiogenic effects on HMEC-1 cells, zebrafish, and matrigel plug mice [87]. Hence, further in-depth and extensive studies need to be performed to demonstrate its potential as an antitumor drug against hepatobiliary cancer.

### 3.4. Bisacurone

Bisacurone (Figure 1(d), C_15_H_24_O_3_, 252.354 g/mol) is more separated from the rhizomes of *C. longa* than its radix [88]. Recent studies have demonstrated the superior pharmacological effects of bisacurone compared to curcuminoids and other active ingredients in *C. longa*. For example, curcumin did not cause significant cell viability at a low concentration of 1 *μ*M, while bisacurone at the same concentration caused high cell viability following ethanol treatment in hepatocytes isolated from SD rats [30]. Additionally, bisacurone was more effective at promoting bile secretion and produced more total bile acids than curcuminoids and ar-turmerone in Wistar rats [71]. A significant decrease in the ALT level in rat serum, which was increased by ethanol administration, was observed after a single intake of bisacurone [32].

Accordingly, bisacurone might exhibit hepatoprotective effects on liver injury induced by ethanol intake and cholestasis. Furthermore, its efficacy could be stronger than that of curcuminoids and ar-turmerone.

## 4. Discussion

In this review, the experimental evidence of *C. longa* and its noncurcuminoid constituents (*β*-elemene, germacrone, ar-turmerone, and bisacurone), their pharmacological activities, and their underlying mechanisms that might contribute to the prevention and treatment of different hepatobiliary diseases, was summarized.

The causative factors resulting in hepatobiliary damage include alcohol, drugs, pesticides, heavy metals, excessive accumulation of fats, viral infections, exposure to toxic chemicals or carcinogens, and abnormalities in the immune system. These triggers might stimulate the emergence of the pathological phenomenon in the hepatobiliary system, such as structural and functional damages of tissues, steatosis, inflammation, fibrosis, and tumor [89]. The hepatobiliary system is vulnerable to oxidative stress. Excessive production of free radicals in the mitochondria, peroxisome, and microsomes of the liver parenchymal cells damages Kupffer cells, endothelial cells, and HSCs, as well as parenchymal cells themselves [10]. In addition, the reaction of bile components with free radicals affects the function of the gall bladder, which can cause some hepatobiliary diseases. Hence, resolving the oxidant/antioxidant imbalance and preventing toxic damage to cells and tissues are important to manage hepatobiliary diseases.

**Figure 2:**
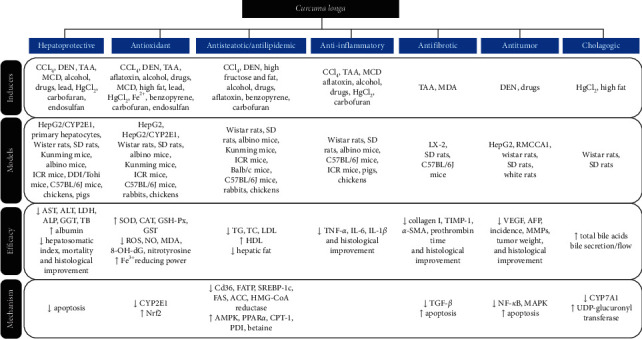
Pharmacological activities of *C. longa* related to hepatobiliary diseases. *C. longa* exhibited hepatoprotective, antioxidant, antisteatotic/hypolipidemic, anti-inflammatory, antifibrotic, antitumor, and cholagogic effects via regulating apoptosis, CYP2E1, Nrf, lipid metabolism-related signaling factors, TGF-*β*, NF-*κ*B, CYP7A1, and so on.

*C. longa* displayed its potency in regulating the oxidant/antioxidant disparity and exhibiting hepatoprotective effects. These pharmacological effects might be activated through the regulation of CYP2E2 and Nrf expression, antiapoptosis, and detoxification of protein generation in microsomes of the liver. *C. longa* significantly altered steatosis, hyperlipidemia, and inflammatory reactions which occurred in the hepatobiliary system. Furthermore, it could markedly suppress fibrosis, tumorigenesis, and cholestasis by inhibiting TGF-*β* signaling, the NF-*κ*B pathway, and CYP7A1 activity (Figure 2).

Based on these preclinical effects of *C. longa*, it can be used as therapeutic candidates for the treatment of toxic hepatitis, alcoholic liver disease, nonalcoholic fatty liver disease, cirrhosis, liver cancer, and cholestatic liver diseases. Regarding the molecular mechanism of *C. longa* in treating hepatobiliary diseases, further investigations on its role as a PPAR-*γ* agonist are needed, which might contribute to regulating inflammation, fibrosis, and cancer. Although the optimal extraction method of *C. longa* is inconclusive, it is necessary to find an extraction process which can maximize the acquisition of its active constituents.

**Figure 3:**
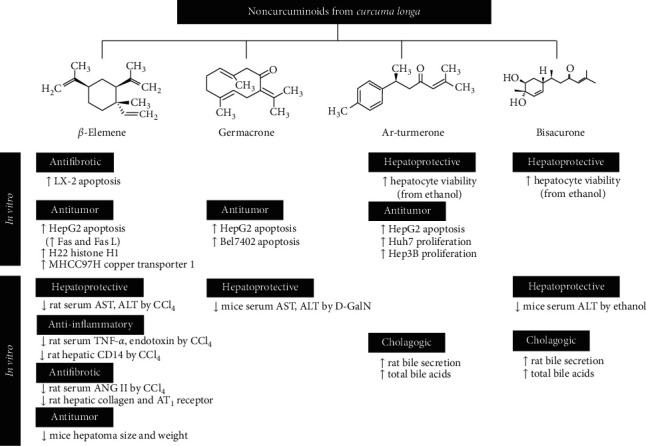
Pharmacological activities of four chemical compounds from *C. longa*. Four sorts of noncurcuminoid ingredients obtained from *C. longa* showed hepatoprotective effects commonly. Among them, *β*-elemene could be an attractive compound to treat different hepatobiliary diseases because it was excellent in anti-inflammatory, antifibrotic, and antitumor activities, as well as hepatoprotective effects.

With regard to the four compounds belonging to the noncurcuminoid group obtained from *C. longa*, the hepatoprotective effects were commonly observed when all four constituents were administered. In particular, ar-turmerone and bisacurone protected hepatic parenchymal cells from alcohol-induced damage. Germacrone and *β*-elemene were found to exhibit hepatoprotective actions against D-GalN and CCl_4_, respectively. In addition, *β*-elemene, germacrone, and ar-turmerone promoted the apoptosis of HepG2 cells, and ar-turmerone inhibited the proliferation of Huh7 and Hep3B cells. The combination of *β*-elemene and conventional anticancer drugs has been suggested because it was found to suppress H22 cell proliferation by activating the histone H1 protein and elevating the sensitivity of anticancer drugs in MHCC97H cells. *β*-Elemene has been approved as a Class II noncytotoxic therapeutic antitumor agent by the China Food and Drug Administration because of its low toxicity, high efficacy, and immune-enhancing effects [90] (Figure 3).

Interestingly, the *C. longa* extracts exhibited various pharmacological effects when administered to treat hepatobiliary diseases, despite the low bioavailability of curcumin, a representative ingredient of *C. longa*. Such finding infers that *C. longa* may contain active compounds, besides curcumin, that contribute to its efficacy. *β*-Elemene might serve as a strong candidate given its hepatoprotective, anti-inflammatory, antifibrotic, and anticancer effects.

## 5. Conclusions

To our knowledge, this is the first review to summarize the therapeutic activities and pharmacological mechanisms of *C. longa* and its four active constituents, namely, *β*-elemene, germacrone, ar-turmerone, and bisacurone, in experimental models that mimic hepatobiliary diseases. This review presents available evidence regarding *C. longa* and may facilitate its use in the clinic to treat hepatobiliary diseases. However, several limitations exist regarding its use. First, its chemical compounds differ according to the cultivation area, harvest time, and the extraction method. Second, because its four ingredients are essential oils obtained from steam distillation, they have low solubility and poor absorption after ingestion like curcuminoids [74]. Therefore, based on this review, more efforts can be dedicated to broadening the range of clinical application of *C. longa* for treating different hepatobiliary diseases, such as the separation of its active ingredients with high bioavailability and efficacy, its standardization, and the establishment of optimal extraction conditions to maximize its effects.
